# Natural Products that Target the NLRP3 Inflammasome to Treat Fibrosis

**DOI:** 10.3389/fphar.2020.591393

**Published:** 2020-12-17

**Authors:** Nan Ding, Bo Wei, Xiaohui Fu, Chuan Wang, Yimou Wu

**Affiliations:** ^1^ Institute of Pathogenic Biology, Hengyang Medical College, Hunan Provincial Key Laboratory for Special Pathogens Prevention and Control, Hunan Province Cooperative Innovation Center for Molecular Target New Drug Study, University of South China, Hengyang, China; ^2^ Research lab of Translational Medicine, Hengyang Medical College, University of South China, Hengyang, China

**Keywords:** herbs, phytochemicals, fibrosis, NLRP3 inflammasome, terpenoids, phenols

## Abstract

Fibrosis is a common pathway followed by different organs after injury, and it can lead to parenchymal scarring, cellular dysfunction, and even organ failure. The NLRP3 inflammasome is a multiprotein complex composed of the sensor molecule NLRP3, the adaptor apoptosis-associated speck-like protein containing a CARD (ASC), and the effector protease caspase-1. Overactivation of the NLRP3 inflammasome triggers the abundant secretion of IL-1β and IL-18, induces pyroptosis, and promotes the release of a swathe of proinflammatory proteins, all of which contribute to fibrogenic processes in multiple organs. In recent years, screening bioactive natural compounds for NLRP3 inhibitors to alleviate fibrosis has gained broad interest from the scientific community because of the associated cost-effectiveness and easy access. In this review, we systematically and comprehensively summarize the natural products, including terpenoids, phenols, and alkaloids, among others, and the plant-derived crude extracts, that have been reported to ameliorate fibrosis *via* inhibiting NLRP3 inflammasome activation and highlight the underlying mechanisms. Among all the compounds, diterpenoids is the most promising candidates for inhibiting NLRP3 inflammasome activation and improving fibrosis, as they possess combined inhibitory effect on NLRP3 inflammasome assembly and NF-κB signaling pathway. All the information may aid in the development of therapeutic strategies for the treatment of fibrotic diseases.

## Introduction

Fibrosis refers to the excessive accumulation of extracellular matrix (ECM) due to persistent or severe tissue injury, aging, or genetic factors that can lead to permanent scarring, organ dysfunction, and even death (Noronha et al., 2002; Wynn and Ramalingam, 2012). Many conditions, including liver, kidney, heart, and lung disorders, chronic autoimmune diseases, and tumors, would ultimately suffer from the pathological manifestation of fibrosis without reasonable intervention.

Abnormal activation of the NLRP3 inflammasome is an important contributor to inflammatory disease and subsequent fibrosis. Numerous reviews have systematically clarified the pivotal role played by the NLRP3 inflammasome in fibrogenic disorders, including those of the liver, kidney, heart, and lung (Pelegrin et al., 2017; Kim et al., 2019; Pinar et al., 2020), which suggests that targeting the NLRP3 inflammasome has potential as a therapeutic strategy to treat fibrosis.

The synthetic compound MCC950 (also known as CRID3 and CP 456,773) is the most studied NLRP3 inhibitor, which specifically suppresses NLRP3 inflammasome assembly to block all NLRP3 activation pathways. However, although MCC950 displays significant efficacy in various NLRP3-dependent murine disease models, the phase-Ⅱ clinic trials for rheumatoid arthritis are unsuccessful owning to elevated serum liver enzyme levels in the clinic (Mangan et al., 2018). Therefore, screening NLRP3 inflammasome inhibitors with low toxicity from natural compounds has become an effective means of identifying drugs to treat inflammation and fibrosis. In this review, we aim to summarize the natural products, including terpenoids, phenols, and alkaloids, among others, that target the NLRP3 inflammasome to ameliorate fibrosis both *in vivo* and *in vitro*. This information may aid in the development of novel strategies for the discovery of antifibrotic drugs.

## The Process of Fibrosis

The occurrence of fibrosis can be divided into four major phases (Rockey et al., 2015). The first is response initiation, during which primary injury occurs and proinflammatory factors are released by injured epithelial and/or endothelial cells. This is followed by the activation of effector cells, during which chemokines and growth factors, such as matrix metalloproteinases (MMPs), interleukin (IL)-13, and transforming growth factor beta 1 (TGF-β1), are secreted. The next phase comprises the elaboration of the ECM, during which myofibroblasts, the key effector cells, proliferate and synthesize abundant ECM under pathological conditions, eventually leading to excessive accumulation of collagen, which represents the fourth phase of fibrosis (Strutz, 1995; Noronha et al., 2002; Rockey et al., 2015). In summary, the essence of fibrosis is the activation of quiescent fibroblasts and the induction of ECM production and accumulation.

ECM is made of various proteins, proteoglycans, and glycosaminoglycans, including the main components collagen and fibronectin. The deposition of ECM depends on the balance between ECM synthesis and degradation. MMPs, the main enzymes responsible for ECM degradation, are able to cleave ECM components with wide substrate specificities. The activity of MMPs is relatively low in normal conditions but improved during repairing or remodeling processes and in diseased or inflamed tissue. The tissue inhibitor of the metalloproteinases (TIMP) family are the main components that reversibly inhibit the activity of MMPs, thus avoiding excessive and deleterious tissue degradation (Bonnans et al., 2014).

## The NLRP3 Inflammasome and Fibrosis

Acute and chronic inflammation are the main fibrosis-triggering factors. Resident epithelial or endothelial cells are often injured under inflammatory conditions, leading to enhanced release of mediators of inflammation, such as cytokines and chemokines. These factors induce the recruitment of a variety of inflammation-related cells, such as macrophages, lymphocytes, eosinophils, and basophils, that activate fibroblasts and myofibroblasts, thereby leading to fibrosis (Rockey et al., 2015).

The NLRP3 inflammasome is the most widely studied of the inflammasomes. It is composed of the sensor molecule NLRP3, the adaptor ASC (apoptosis-associated speck-like protein containing a CARD), and the effector protease caspase-1 (procaspase-1). The NLRP3 inflammasome can recognize pathogen-associated molecular patterns (PAMPs) and danger-associated molecular patterns (DAMPs), which triggers the cleavage of dormant procaspase-1 to generate active caspase-1, further converting the cytokine precursors pro-IL-1β and pro-IL-18 into mature and biologically active IL-1β and IL-18, respectively (Schroder and Tschopp, 2010). In addition, the caspase-1 also can cleave the gasdermin D (GSDMD), a complex composed of an amino-terminal cell death domain (GSDMD^Nterm^), a central short linker region, and a carboxy-terminal autoinhibition domain, to release the GSDMD^Nterm^, which combines with phosphatidylserine and phosphatidylinositol phosphates in the cell membrane inner leaflet, further forming a 10–14 nm pore containing 16 symmetrical protomers, thus leading to the death of cells and excessive release of inflammatory mediators, including IL-1β and IL-18 (Swanson et al., 2019).

The expression of NLRP3 and associated proteins is markedly enhanced in the organs of fibrosis patients (Wree et al., 2014; Ke et al., 2018). Persistent activation of the NLRP3 inflammasome leads to the abundant secretion of IL-1β and IL-18 and initiation of pyroptosis, all of which are major contributors to the formation of fibrotic lesions (Pinar et al., 2020). IL-1β is a potential mediator of the crosstalk between inflammation and fibrosis and can generate a positive feedback cycle to dominate the secretory cytokine profile in cystic fibrosis (Otto, 2018; Chen et al., 2019). Additionally, the deletion or inhibition of IL-18 can alleviate cardiac or renal fibrosis in corresponding mouse models (Liang et al., 2018; Xiao et al., 2018). Several mechanisms underly how IL-1β, IL-18, and pyroptosis enhance inflammation and fibrosis after inflammasome activation. First, secreted IL-1β and IL-18 bind to the IL-1 receptor, type I (IL-1RI) and IL-18R, which further activates the nuclear factor kappa B (NF-κB) signaling pathway to induce the expression of multiple inflammation-related proteins. Second, following the release of proteins such as caspase-1 and nuclear protein high-mobility group box 1 (HMGB1) into the extracellular space, the immune system will detect the presence of these types of proteins and activate inflammatory responses. Moreover, the occurrence of pyroptosis promotes the release of various protein complexes, especially those comprising the NLRP3 inflammasome. On the one hand, extracellularly localized inflammasomes can activate caspase-1 owing to the presence of high concentrations of procaspase-1 in the extracellular milieu, thereby inducing a series of inflammatory responses. On the other hand, macrophages can internalize extracellular inflammasomes, thus further promoting caspase-1 activation (Baroja-Mazo et al., 2014; Pelegrin et al., 2017). The enhanced secretion of IL-1β is able to downregulate MMPs, upregulate TIMPs and promote the production of collagen I (Pinar et al., 2020). IL-18 also has been shown to possess an 8-fold enhancement in interstitial collagen content, leading to increased ECM accumulation and fibrogenesis (O'|’Brien et al., 2014). Additionally, the augmented inflammasome-independent NLRP3, IL-1β and IL-18 all can activate the mitogen-activated protein kinase (MAPK) signaling pathway to upregulate the expression of TGF-β1 and induce fibroblast differentiation and collagen production (Artlett, 2012; Wang et al., 2013).

## NLRP3 Inflammasome Activation

**FIGURE 1:**
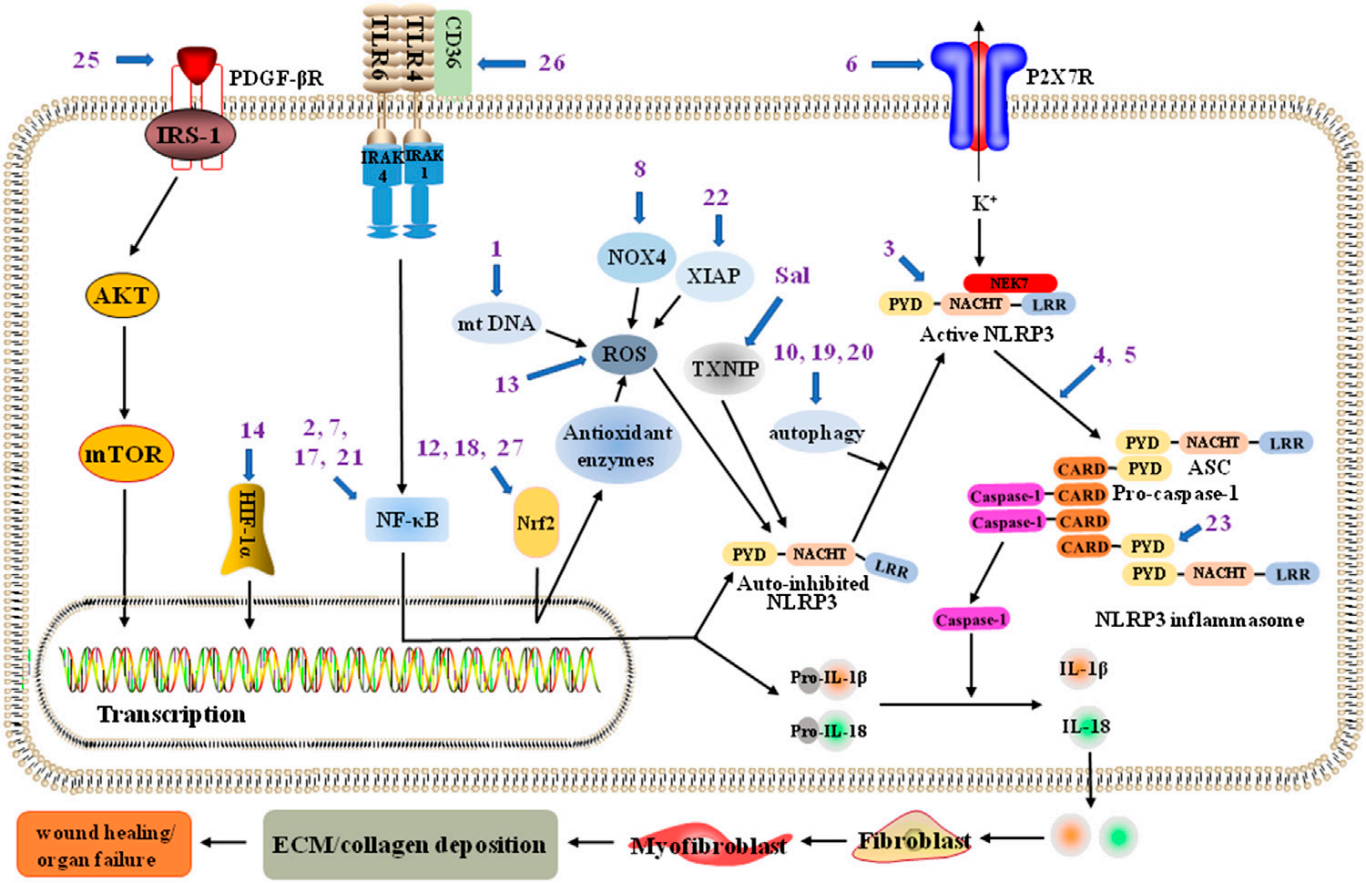
The NLRP3 inflammasome pathway associated with potential blockade sites of various pharmacological inhibitors to treat fibrosis. NLRP3 (NOD-, LRR- and pyrin domain-containing 3) inflammasome in liver fibsosis could be inhibited by NLRP3 inflammasome assembly inhibitors andrographolide (**4**) and oridonin (**3**, targeting the NACHT domain in NLRP3), mitochondrial DNA (mt DNA) *de novo* synthesis inhibitor sweroside (**1**), P2X purinoceptor 7 (P2X7) inhibitor 25-OCH_3_-PPD (**6**), Nrf2 pathway promotor quercetin (**12**) and pelargonidin (**18**), and platelet-derived growth factor-*β* receptor (PDGF-*β*R) inhibitor tetramethylpyrazine (**25**); NLRP3 inflammasome in renal fibsosis could be inhibited by nuclear factor (NF)-κB pathway inhibitors artemisinin (**2**), compound K (**7**), and icariin (**17**), Nrf2 pathway promotor astaxanthin (**27**), nicotinamide adenine dinucleotide phosphate oxidase 4 (NOX4) inhibitor ginsenoside Rg1 (**8**), ROS production inhibitor dihydroquercetin (**13**), autophagy activator pterostilbene (**20**), and X-linked inhibitor of apoptosis protein (XIAP) suppressant (**22**); NLRP3 inflammasome in cardiac fibsosis could be inhibited by NLRP3 inflammasome assembly inhibitor triptolide (**5**), CD36 inhibitor cinnamaldehyde (**26**), and TXNIP suppressant of crude extract salvianolates (Sal); NLRP3 inflammasome in pulmonary fibsosis could be inhibited by NLRP3 inflammasome assembly inhibitor lycorine (**23**, targeting the PYD of ASC), autophagy activator resveratrol (**19**), and NF-κB pathway inhibitor polydatin (**21**); NLRP3 inflammasome in synovial tissue fibsosis could be inhibited by hypoxia-inducible factors (HIF)-1α pathway inhibitor casticin (**14**); NLRP3 inflammasome in pancreatic fibrosis might be inhibited by autophagy activator saikosaponin A (**10**).

NLRP3 inflammasome activation consists of two steps: priming and activation. In the resting state, the amount of NLRP3 present in the cell is insufficient to allow inflammasome activation. In the priming step, the NF-κB pathway is activated by pattern recognition receptors (PRRs) such as Toll-like receptors (TLRs), nucleotide-binding oligomerization domain-containing protein 2 (NOD2), and the granulocyte–macrophage colony stimulating factor (GM–CSF) receptor; this leads to the upregulation of the transcription and expression of NLRP3 and other key proinflammatory components, including pro-IL-1β and pro-IL-18 (Shao et al., 2019). The expressed NLRP3 undergoes autoinhibition due to phosphorylation, dephosphorylation, ubiquitination, or alkylation modifications, which work together to regulate NLRP3 degradation and self-association. NLRP3 is subsequently activated by processes such as K^+^ efflux, Ca^2+^ flux, Cl^−^ efflux, lysosomal disruption, and mitochondrial dysfunction (ROS production), which triggers the assembly of the NLRP3, ASC, and procaspase-1 proteins into the NLRP3 inflammasome. Mature caspase-1 is then released, resulting in further rapid processing of pro-IL-1β and pro-IL-18 into biologically active IL-1β and IL-18, respectively (Swanson et al., 2019) (Figure 1). Additionally, the NLRP3 inflammasome can also be activated *via* a noncanonical signaling pathway in which caspase-11cleave the GSDMD and induce pyroptosis, thus promoting K^+^ efflux and the release of IL-1β (Kayagaki et al., 2015; Wang et al., 2020a).

## Phytochemicals That Suppress the NLRP3 Inflammasome and Inhibit Fibrosis

**FIGURE 2:**
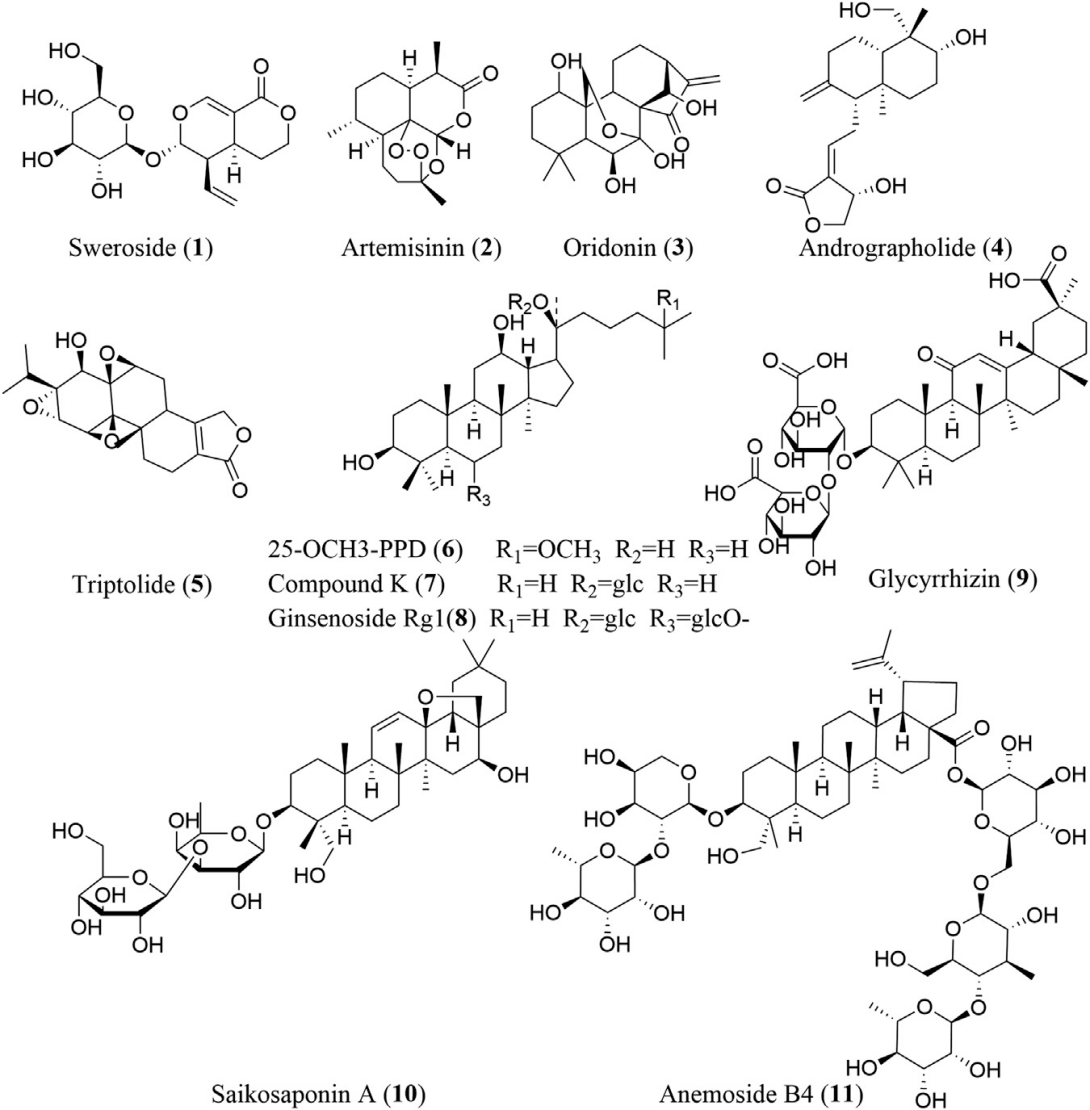
The chemical structures of terpenoids that inhibit NLRP3 inflammasome activation.

**FIGURE 3:**
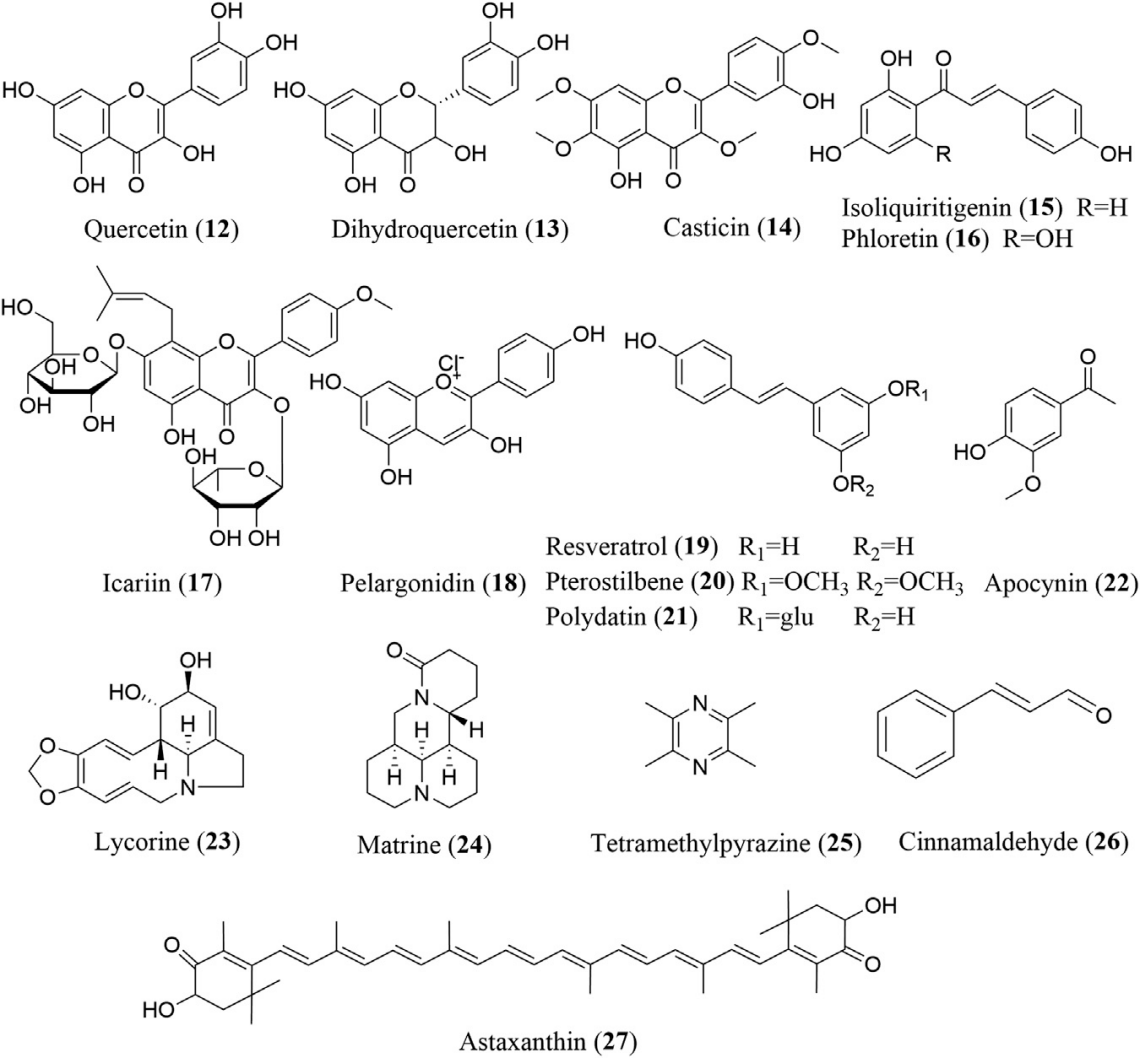
The chemical structures of phenols, alkaloids, and other compounds that inhibit NLRP3 inflammasome activation.

Screening for NLRP3 inhibitors from natural products to treat fibrosis has gained broad interest from the scientific community. Numerous studies have reported that natural products, including terpenoids (Figure 2), phenols, alkaloids, and other compounds (Figure 3), exhibit significant inhibitory activity against the NLRP3 inflammasome and can alleviate various fibrotic disorders, both *in vitro* and *in vivo*. Here, we describe all the phytochemicals that have been reported to ameliorate fibrosis *via* NLRP3 inflammasome inhibition in various cell and animal models, as well as the mechanisms underlying these inhibitory effects (Table 1).

**TABLE 1:** The phytochemicals reported to inhibit NLRP3 inflammasome activation in various fibrotic diseases.

No	Phytochemicals	Sources	*In vitro*	*In vivo*
**1**	Sweroside (Gousiadou et al., 2015; Yang et al., 2020)	*Manulea altissima* (Scrophulariaceae)	Inhibiting activation of NLRP3 inflammasome and formation of ASC speck in BMDMs	Blocking the *de novo* synthesis of mtDNA to suppress NLRP3 inflammasome activation in the liver of NASH C57BL/6 mice
**2**	Artemisinin (Wen et al., 2019)	*Artemisia annua* L	Downregulating the NF-κB/NLRP3 pathway in HK-2	Downregulating the NF-κB/NLRP3 pathway in SNx Sprague-Dawley rats
**3**	Oridonin (He et al., 2018)	*Rabdosia rubescens*	Targeting the NACHT domain in NLRP3 *via* combining with the cysteine 279 to block NLRP3-NEK7 interaction in BMDMs Inhibiting TGF-β1 enhanced NLRP3 inflammasome activation in HSCs	Reducing the NLRP3 inflammasome activation in C57BL/6J mice with CCl_4_-induced liver fibrosis
**4**	Andrographolide (Cabrera et al., 2017)	*Andrographis paniculata*	Inhibiting NF-κB pathway and NLRP3 inflammasome assembly in fat-laden HepG2 cells	Suppressing NLRP3 inflammasome activation in C57BL/6 mice with alcoholic steatohepatitis to improve liver fibrosis
**5**	Triptolide (Li et al., 2017b; Pan et al., 2019)	*Tripterygium wilfordii* Hook F	Interrupting NLRP3/ASC interaction to reduce the AngII-triggered collagen production in CFs	Alleviating chronic pressure-overloaded or isoproterenol-induced cardiac fibrosis by inhibiting NLRP3/TGFβ1/Smad signaling in C57BL/6 mice
**6**	25-OCH3-PPD (Han et al., 2018)	*Panax notoginseng*	Promoting activity of LXRs to ameliorate P2X7R-mediated NLRP3 inflammasome activation to decrease *a*-SMA expression in HSC-T6	Affecting P2X7R to suppress NLRP3 inflammasome activation in TAA-stimulated liver fibrosis in C57BL/6 mice
**7**	Compound K (Hsu et al., 2020)	Metabolite of ginsenoside Rb1	Inhibiting the priming and mitochondria-mediated NLRP3 inflammasome activation in TECs and J774A.1 macrophages	Targeting the NF-κB/NLRP3 pathway to improve fibrosis in C57BL/6 mice with renal tubulointerstitial lesions
**8**	Ginsenoside Rg1 (Shen et al., 2020)	Ginseng	NA	Decreasing NOX4-induced ROS production and the subsequent NLRP3 inflammasome activation in SAMR1 and SAMP8 mice
**9**	Glycyrrhizin (Yan et al., 2018)	*Glycyrrhiza glabra*	Inhibiting deoxycholic acid-triggered NLRP3 inflammasome activation in raw 264.7	Decreasing bile acids accumulation to suppress NLRP3 inflammasome activation in C57BL/6 mice with MCD diet-induced NASH
**10**	Saikosaponin A (Cui et al., 2020b)	*Bupleurum falcatum*	Inhibiting AMPK/mTOR-mediated autophagy and NLRP3 inflammasome activation in pancreatic stellate cells	NA
**11**	Anemoside B4 (Gong et al., 2019)	Pulsatilla radix	NA	Reducing the fiber collagen and expression of NLRP3, caspase-1, ASC, IL-1β, and IL-18 in adenine-induced renal injury Wistar rats
**12**	Quercetin (Choe and Kim, 2017; Liu et al., 2018)	Multiple plants	Regulating TXNIP to suppress ROS production and NLRP3 inflammasome activation in fructose-induced U937 and THP-1 macrophages	Activating Nrf2 to upregulate expression of OH-1 to modulate ROS/NF-κB/NLRP3 pathway in Wistar rats
**13**	Dihydroquercetin (Ding et al., 2018)	*Larix sibirica* (Pinaceae) and *Pseudotsuga taxifolia* (Pinaceae)	Suppressing mitochondrial ROS generation and NLRP3 inflammasome activation in high glucose stimulated HBZY-1 and HK2 cells	Decreasing ROS production and NLRP3 inflammasome activation to improve fibrosis in SD rats with HFD/streptozotocin-induced diabetic nephropathy
**14**	Casticin (Li et al., 2020)	Vitex fructus	Suppressing NLRP3 components in primary synovial fibroblasts	Targeting HIF-1α/NLRP3 inflammasome pathway in MIA-induced knee osteoarthritis of SD rats
**15**	Isoliquiritigenin (Peng et al., 2015; Watanabe et al., 2016)	Several plants of the genus *Glycyrrhiza*	Inhibiting NLRP3 inflammasome activation in raw 264.7 and 3T3-L1 cells	Inhibiting NLRP3 inflammasome and PPAR-γ activation in the adipose tissue of HFD-fed C57BL/6 mice
**16**	Phloretin (Cui et al., 2020a)	Apples and strawberries	Repressing NLRP3 components in HK-2 cells	Co-inhibiting NLRP3 inflammasome activation and uric acid reabsorption inhyperuricemia-induced renal fibrosis of C57BL/6 mice
**17**	Icariin (Zhang et al., 2017)	Several species of the genus *Epimedium*	NA	Inhibiting the NF-κB and NLRP3 signaling pathways in BGG-induced IgAN of Sprague–Dawley rats
**18**	Pelargonidin (Shi et al., 2020)	Red geraniums and berries	Activating Nrf2 to attenuate cellular oxidative stress and suppress NLRP3 inflammasome activation in TGF-β-challenged HSCs	Suppressing the ROS/NLRP3/il-1β axis *via* activating the Nrf2 pathway in In CCl_4_-induced C57BL/6J mice
**19**	Resveratrol (Ding et al., 2019)	Wine and grapes	Inhibiting autophagy to attenuate particulate matter (PM_2.5_)-induced cytotoxicity in PM2.5-treated BEAS-2B cells	Downregulating the autophagy-related NLRP3 inflammasome activation in PM_2.5_-triggered lung fibrosis of BALB/c mice
**20**	Pterostilbene (Wang et al., 2020d)	Multiple foods and herbs	Augmenting autophagy to inhibit NLRP3 inflammasome activation in TGF-β stimulated NRK-52E cells	Enhancing autophagy to restrain EMT-mediated NLRP3 inflammasome activation in PO-induced hyperuricemia or HAD-triggered CKD of ICR mice
**21**	Polydatin (Tang et al., 2019)	*Polygonum cuspidatum*	Inhibiting fibrotic markers in BEAS-2B cells	Suppressing activation of NF-κB/NLRP3 signaling pathway in mp- infected BALB/c mice
**22**	Apocynin (Xin et al., 2018)	*Picrorhiza kurroa*	NA	Inhibiting NLRP3/XIAP signaling pathway of Sprague-Dawley rats with DN
**23**	Lycorine (Liang et al., 2020)	Amaryllis and daffodil	Targeting the PYD of ASC on Leu9, Leu50 and Thr53 to interrupt NLRP3-ASC interaction in BMDMs	Inhibiting NLRP3 inflammasome activation and pyroptosis in bleomycin-induced pulmonary fibrosis of C57BL/6 mice
**24**	Matrine (Mahzari et al., 2019)	Plants of the genus *Sophora*	NA	Decreasing fibrotic markers partially *via* inhibiting NLRP3 inflammasome activation in NASH C57BL/6J mice
**25**	Tetramethylpyrazine (Zhang et al., 2014; Wu et al., 2015)	*Ligusticum wallichii* Franch	Targeting PDGF-βR/NLRP3/caspase-1 pathway in PDGF or Angiotensin II treated HSCs	Targeting PDGF-βr/NLRP3/caspase-1 pathway in CCl_4_-induced liver fibrosis of Sprague-Dawley rats
**26**	Cinnamaldehyde (Kang et al., 2016)	Species of the genus *Cinnamomum*	Repressing NLRP3 inflammasome activation *via* CD-36-mediated TLR4/6-IRAK4/1 pathway in fructose-exposed H9c2 cells	Attenuating CD-36 mediated TLR4/6-IRAK4/1 signaling to inhibit NLRP3 inflammasome activation in fructose-induced cardiac fibrosis of Sprague-Dawley rats
**27**	Astaxanthin (Liu et al., 2015)	Microalgae and seafood	NA	Promoting Nrf2 expression to inhibit NLRP3 inflammasome activation in doxorubicin-induced focal segmental glomerulosclerosis of BALB/c

### Terpenoids

Terpenoids are a large group of widely distributed, bioactive natural products with diverse structures, and are composed of five-carbon (C5) units such as isopentenyl diphosphate or dimethylallyl diphosphate. Four main types of terpenoids have been reported to inhibit NLRP3 inflammasome activation: monoterpenoids (C10), sesquiterpenoids (C15), diterpenoids (C20), and triterpenoids (C30).

#### Monoterpenoids

Sweroside (**1**) is an iridoid monoterpene glucoside with obvious antifibrotic activity. It can effectively inhibit NLRP3 inflammasome activation in bone marrow-derived macrophages (BMDMs). Additionally, in a mouse model of nonalcoholic steatohepatitis (NASH) induced by a methionine–choline-deficient (MCD) diet, sweroside significantly attenuates NASH symptoms (including liver fibrosis) through the suppression of NLRP3 inflammasome activation in the liver, partially by inhibiting *de novo* synthesis of mitochondrial DNA (mtDNA), which can be oxidized to serve as the ultimate NLRP3 ligand, or at least a part of it, thus activating NLRP3 inflammasome complexes after exposure to LPS and NLRP3 activators (Zhong et al., 2018; Yang et al., 2020).

#### Sesquiterpenoids

Artemisinin (**2**) is a sesquiterpene endoperoxide known primarily as an effective medicine for the treatment of malaria. However, artemisinin and its derivatives have also shown potential antifibrotic value in recent studies (Wang et al., 2020c). Artemisinin can downregulate the NF-κB/NLRP3 pathway to attenuate tubulointerstitial fibrosis in subtotal nephrectomized (SNx) rats, which is consistent with the results observed in Ang II-treated human kidney 2 (HK-2) cells (Wen et al., 2019).

#### Diterpenoids

Oridonin (**3**), andrographolide (**4**), and triptolide (**5**) are three diterpenoids exhibiting an antifibrotic effect *via* inhibiting NLRP3 inflammasome activation. In LPS-primed and nigericin-stimulated BMDMs, oridonin targets the NACHT domain of NLRP3 by binding to cysteine 279, thereby blocking NLRP3-NEK7 (a mitotic Ser/Thr kinase bridging adjacent NLRP3 subunits with bipartite interactions to activate the NLRP3 inflammasome) interaction and inhibiting NLRP3 inflammasome assembly and activation (He et al., 2018; Sharif et al., 2019). However, the specificity of oridonin for NLRP3 is dose-dependent, for it is insensitive to lipopolysaccharide (LPS)-induced pro-IL-1β, NLRP3 expression and TNF-α production but effective to inhibit the level of IL-1β at doses between 0.5 and 2.0 μM (He et al., 2018). In addition, oridonin also inhibits TGF-β1 enhanced NLRP3 inflammasome activation in a human HSCs cell line, LX-2. Andrographolide can suppress LPS-induced IL-1β expression through NF-κB inhibition in fat-laden HepG2 cells and inflammasome disassembly (Cabrera et al., 2017). Triptolide exhibits significant efficacy in reducing the AngII-induced collagen production in cardiac fibroblasts by interrupting NLRP3/ASC interaction to inhibit NLRP3 inflammasome activation (Pan et al., 2019).

A series of research has also confirmed the efficacy of the diterpenoids above *in vivo*. In mice with CCl_4_-induced liver fibrosis, oridonin treatment significantly reduces the collagen deposition, HSCs (cells associated with hepatic fibrosis) activation, and recruitment of Kupffer cells, which may be related to its inhibitory effect on NLRP3 inflammasome activation in HSCs, highlighting the potential of the compound for use in the treatment of liver fibrosis. In mice with alcoholic steatohepatitis, andrographolide improves the liver fibrosis, decreases the hepatic fibrogenic proteins, such as connective tissue growth factor, collagen type I, alpha 1 chain and collagen IV, and suppresses the NLRP3 inflammasome activation (Cabrera et al., 2017). Recent studies have demonstrated the significant ability of triptolide to alleviate cardiac fibrosis through NLRP3 inflammasome inhibition. In chronic pressure-overloaded mice treated with triptolide, myocardial remodeling was attenuated through inhibition of the NLRP3 inflammasome and subsequently downstream inflammatory mediators such as IL-1β and IL-18, as well as inhibition of the TGF-β1 pathway (Li et al., 2017b). Additionally, triptolide alleviates isoproterenol-induced cardiac fibrosis by inhibiting NLRP3/TGFβ1/Smad signaling in the mouse ventricle (Pan et al., 2019).

#### Triterpenoids

A myriad of triterpenoids and their derivatives, including the dammarane triterpenoids 25-OCH_3_-PPD (**6**), compound K (**7**), and ginsenoside Rg1 (**8**); the oleanane triterpenoid saponins glycyrrhizin (**9**) and saikosaponin A (**10**); and the lupane triterpenoid saponin anemoside B4 (**11**), exhibit markedly antifibrotic activity *via* inhibiting NLRP3 inflammasome activation.

*In vitro* study reveals that 25-OCH_3_-PPD can decrease the expression of alpha smooth muscle actin (α-SMA) in TGF-β stimulated HSC-T6 cell *via* inhibiting NLRP3 inflammasome assembly, which is dependent on the inhibition of the purinergic 2 X7 receptor (P2X7R) induced by the promotion of liver X receptors (LXRs) (Han et al., 2018). Compound K possesses an inhibitory effect on the priming and mitochondria-mediated activation of the NLRP3 inflammasome as well as the STAT3 signaling pathway in renal tubular epithelial cells (TECs) under mechanically induced constant pressure (MICP) and murine J774A.1 macrophages. In Raw 264.7 macrophage cells, both glycyrrhizin and its metabolite glycyrrhetinic acid can significantly inhibit deoxycholic acid-induced NLRP3 inflammasome activation. Saikosaponin A exerts distinct inhibitory effects in suppressing the activation of pancreatic stellate cells, which are vital for the development of pancreatic fibrosis, by inhibiting autophagy and the NLRP3 inflammasome activation *via* the AMPK/mTOR pathway (Cui et al., 2020b).

Various studies have demomstrated the anti-fibrotic efficacy of triterpenoids in mice. Both 25-OCH_3_-PPD and glycyrrhizin are effective in inhibiting hepatic fibrosis. In thioacetamide (TAA)-stimulated liver fibrosis of mice, 25-OCH_3_-PPD possesses the ability to reduce the expression levels of TIMP-1, MMP-13, and collagen I as well as the inhibition of ECM deposition, which is partially due to the regulation of NLRP3 inflammasome signaling pathway by affecting P2X7R activation (Han et al., 2018; Su et al., 2019). In mice with MCD diet-induced NASH, glycyrrhizin and its metabolite glycyrrhetinic acid can significantly decrease bile acids accumulation, thus inhibiting the activation of NLRP3 inflammasome to attenuate liver fibrosis (Yan et al., 2018). Compound K, ginsenoside Rg1, and anemoside B4 are reported to exhibit inhibitory effect on renal fibrosis. In mice with renal tubulointerstitial lesions, compound K reduces collagen Ⅲ and total collagen content in the tubulointerstitial compartment, most likely by targeting the NF-κB/NLRP3 pathway (Hsu et al., 2020). Ginsenoside Rg1 can delay kidney aging and inhibit aging-related glomerular fibrosis by decreasing NOX4-induced ROS production and subsequently downregulating the expression of NLRP3 inflammasome-associated proteins and production of the proinflammatory factor IL-1β (Shen et al., 2020). Anemoside B4 can reduce fiber collagen levels in the renal interstitium, lower the expression of NLRP3, caspase-1, ASC, IL-1β*,* and IL-18, and enhance the expression of podocin and nephrin (two major podocyte proteins), suggesting that it exerts a protective effect on adenine-induced renal injury in rats (Gong et al., 2019).

### Phenols

Phenolic compounds are secondary metabolites bearing one or more hydroxyl groups in the aromatic ring of their structures and are widely found in plants such as vegetables, fruits, and tea. Phenolic compounds are classified into multiple groups, including flavonoids, phenolic acids, and stilbenes, based on their structural characteristics (Lima et al., 2019).

#### Flavonoids

Flavonoids are naturally occurring and widely distributed plant secondary metabolites, and exhibit numerous biological functions, including antioxidative, antimicrobial, anti-inflammatory, cardioprotective, and antifibrotic activities. The anti-inflammatory effect of flavonoids is mainly due to their ability to inhibit the production of proinflammatory mediators, such as TNF-α, IL-1β, IL-6, and IL-18, and regulate inflammation-related signaling pathways, such as the TLR, NF-κB, MAPK, and AP-1 pathways (Yi, 2018). An increasing number of flavonoids have been reported to exhibit anti-inflammatory and antifibrotic activities by inhibiting NLRP3 inflammasome activation, mainly including quercetin (**12**), dihydroquercetin (**13**), casticin (**14**), isoliquiritigenin (**15**), phloretin (**16**), icariin (**17**), and pelargonidin (**18**).

Pelargonidin, a type of anthocyanidin, can activate nuclear factor erythroid 2-like factor 2 (Nrf2) to attenuate cellular oxidative stress, thus suppressing the NLRP3 inflammasome activation and decreasing the COL1A1, TIMP1 production in TGF-β-challenged HSCs, suggesting that Nrf2-mediated ROS/NLRP3/IL-1β signaling is involved in the anti-fibrotic effect of pelargonidin (Shi et al., 2020). Dihydroquercetin, a dihydroflavone compound, has been reported to attenuate the expression of renal fibrosis-associated proteins fibronectin and collagen IV in high glucose stimulated rat kidney mesangial cells (HBZY-1) and HK-2 cells, which may be associated with the suppression of mitochondrial ROS generation as well as NLRP3 inflammasome activation (Ding et al., 2018). Additionally, the naturally occurring dihydrogen chalcone flavonoid phloretin also inhibits the expression of NLRP3 as well as cleaved caspase-1, IL-1β and IL-18 protein in HK-2 cells. Quercetin is a naturally occurring dietary flavonoid and acts as a supplement with excellent safety and bioavailability (Harwood et al., 2007). *In vitro* studies demonstrate that quercetin can suppress ROS production and subsequent NLRP3 inflammasome activation in fructose-induced U937 and THP-1 macrophages *via* the regulation of intracellular TXNIP, a protein that binds with NLRP3 to promote NLRP3 inflammasome activation (Choe and Kim, 2017). Casticin, also known as vitexicarpin, is effective at suppressing inflammatory factors (IL-1β and IL-18) and fibrogenic markers (TGF-β, COL1A1, and TIMP-1) *via* inhibiting NLRP3 components in primary synovial fibroblasts. Isoliquiritigenin, a flavonoid with a chalcone structure, inhibits TLR4-or Mincle-induced expression of fibrosis-associated genes in stromal vascular fraction from obese adipose tissue and macrophages *in vitro via* targeting the NLRP3 inflammasome.

The flavonoids also exhibit significantly anti-fibrotic effects in various disease models. Both quercetin and pelargonidin contribute to alleviating hepatic fibrosis by upregulating Nrf2 to inhibit NLRP3 inflammasome activation. In rats with acute alcoholic liver injury (AALI), quercetin activates Nrf2 to upregulate the expression of heme oxygenase-1 (HO-1), thus leading to a reduction in NLRP3 inflammasome activity and secretion of IL-1β and IL-18 (Liu et al., 2018). In CCl_4_-induced mice, pelargonidin suppress *a*-SMA and COL1A1 protein expression in liver *via* increasing Nrf2 levels and reducing oxidative stress, thereby inhibiting the NLRP3 inflammasome (Shi et al., 2020). Dihydroquercetin, phloretin, and icariin are all effective in ameliorating NLRP3 inflammasome-involved renal fibrosis. Dihydroquercetin treatment can decrease ROS production and NLRP3 inflammasome activation to improve fibrosis in rats with high fat diet (HFD)/streptozotocin-induced diabetic nephropathy (DN) (Ding et al., 2018). Phloretin can reduce hyperuricemia-induced renal inflammation, fibrosis, and mitochondrial stress in mice by co-inhibiting NLRP3 inflammasome activation and uric acid reabsorption (Cui et al., 2020a). In rats with bovine gamma globulin (BGG)-induced IgA nephropathy (IgAN), icariin greatly decreases IgA deposition and glomerular fibrosis probably by inhibiting the NF-κB and NLRP3 signaling pathways (Zhang et al., 2017). In addition, Casticin exerts significant role in suppressing fibrogenic markers (TGF-β, COL1A1, and TIMP-1) and improving hypoxia in synovial tissue of rats with monoiodoacetic acid (MIA)-induced knee osteoarthritis (KOA) through the modulation of the hypoxia-inducible factor-1α (HIF-1α), which is a transcriptional factor served to promote NLRP3 inflammasome activation (Li et al., 2020). What is more, isoliquiritigenin can ameliorate inflammation and fibrosis in the adipose tissue of HFD-fed mice, and this effect is correlated with the inhibition of the NLRP3 inflammasome and PPAR-γ activation (Watanabe et al., 2016).

#### Stilbenes

Stilbenes, comprising a subclass of polyphenolic compounds, possess many beneficial pharmacological properties, including neuroprotective, cardiovascular protective, antioxidative, and anti-inflammatory effects. Among the stilbenes, resveratrol (**19**), pterostilbene (**20**), and polydatin (**21**) have been reported to exert antifibrotic effects through the regulation of NLRP3 inflammasome activation.

Resveratrol, the most widely studied stilbene, possesses the ability to attenuate particulate matter (PM_2.5_)-induced cytotoxity in PM_2.5_-treated BEAS-2B cells *via* inhibiting autophagy, which is an evolutionarily conserved cellular process facilitating the clearance of NLRP3 activators such as β-amyloid and damaged mitochondria, thus suppressing the NLRP3 inflammasome activation (Nakahira et al., 2011; Cho et al., 2014). Pterostilbene, a dimethylated analog of resveratrol, can also markedly augment autophagy to inhibit NLRP3 inflammasome activation, thereby reducing the TGF-β triggered expression of EMT markers including E-cadherin, fibronectin and *α*-SMA in immortalized rat kidney proximal tubular epithelial cells (NRK-52E cells) (Wang et al., 2020d). Polydatin, a resveratrol glucoside, is able to decrease the expression of *a*-SMA, collagen Ⅰ, and Ⅲ in human lung epithelial BEAS-2B cells, however, the inhibitory effect of which can be reversed by overexpression of NLRP3.

The stilbenes also improve organ fibrosis *in vivo*. In models of potassium oxonate (PO)-induced hyperuricemia and high adenine diet (HAD)-induced chronic kidney disease (CKD), pterostilbene-triggered autophagy has an attenuating effect on the improvement of renal function and interstitial fibrosis *via* restraining EMT-mediated NLRP3 inflammasome activation (Wang et al., 2020d). Resveratrol is effective at alleviating lung inflammation and fibrosis caused by particulate matter (PM_2.5_) *via* inhibiting autophagy-related NLRP3 inflammasome activation in BALB/c mice (Ding et al., 2019). In mice infected with *Mycoplasma pneumomiae*, polydatin treatment can suppress NF-κB/NLRP3 signaling pathway activation, thereby attenuating lung inflammation and fibrosis.

#### Other Phenolic Compounds

Apocynin (**22**), a naturally occurring methoxy-substituted catechol has demonstrated an antifibrotic efficacy in major organs. Apocynin is widely used as an antioxidant *via* the inhibition of NOXs and the generation of superoxide anion, a ROS precursor (Touyz, 2008). Another study showed that apocynin hinders fibrosis in the renal cortex of rats with DN and decreases the expression of NLRP3 and X-linked inhibitor of apoptosis protein (XIAP) (Xin et al., 2018). XIAP functions as a caspase inhibitor, the expression of which is also decreased by the grape seeds with antioxidant activity, suggesting that XIAP may be involved in the NLRP3 inflammasome activation and renal fibrosis by regulating ROS (Guclu et al., 2015).

### Alkaloids

Alkaloids are a group of nitrogen-containing organic secondary metabolites with diverse structures that are extensively distributed in plants, microbes, and even animals. Alkaloids have a wide variety of bioactive functions, including antitumor, antioxidant, anti-inflammatory, and immunoregulatory activities (Liu et al., 2019). Among the alkaloids, lycorine (**23**), matrine (**24**), and tetramethylpyrazine (**25**) have been shown to exert antifibrotic effects *via* inhibiting NLRP3 inflammasome activation.

Lycorine is an Amaryllidaceae alkaloid first identified as a potential candidate to treat cardiac fibrosis (Schimmel et al., 2020). It targets the pyrin domain (PYD) of ASC at Leu9, Leu50, and Thr53 to interrupt the interaction between NLRP3 and ASC, thus inhibiting NLRP3 inflammasome activation and pyroptosis in LPS-primed, nigericin-stimulated BMDMs (Liang et al., 2020). Tetramethylpyrazine, another important alkaloid to treat fibrosis, exerts the ability to suppress the enhanced expression of *a*-SMA and α1(I) procollagen in Angiotensin II-triggered HSCs, which is associated with the PDGF-mediated fibrotic pathways (Zhang et al., 2014). Additionally, tetramethylpyrazine exhibits inhibitory effect on the augmented expression of pro-IL-1β, pro-IL-18, and cleaved IL-1β in platelet-derived growth factor (PDGF)-treated HSCs *via* regulating PDGF beta receptor (PDGF-βR)/NLRP3/caspase-1 pathway (Wu et al., 2015).

Various *in vivo* studies have demonstrated the role of alkaloids in treating fibrosis. In NASH mice fed an MCD diet, matrine decreases the expression of TGF-β, collagen Ⅰ, and Smad3 by inhibiting the NLRP3 inflammasome, at least partially (Mahzari et al., 2019). In CCl_4_-induced liver injury in rats, tetramethylpyrazine possesses an antifibrotic role in alleviating liver injury and fibrogenesis through the involvement of PDGF-βR/NLRP3 signaling pathway. Lycorine is effective at decreasing the *a*-SMA, fibronectin and collagen content in bleomycin-induced pulmonary fibrosis *via* inhibiting NLRP3 inflammasome activation and pyroptosis (Liang et al., 2020).

### Other Compounds

Cinnamaldehyde (**26**) reduces the cellular TGF-β, *p*-Smad2/3 and Smad4 expression in fructose-exposed H9c2 cells by repressing oxidative stress and ROS production, thereby inhibiting NLRP3 inflammasome activation *via* the CD-36-mediated TLR4/6-IRAK4/1 signaling pathway. Cinnamaldehyde protects human dental pulp cells against oxidative stress through the Nrf2/HO-1-dependent antioxidant response. Recent studies have demonstrated the pivotal role of Nrf2 in mediating the antioxidant effect of cinnamaldehyde, which significantly promotes Nrf2 activation, thereby inhibiting the TGF-β1 and IL-13-dependent expression of periostin in fibroblasts (Mitamura et al., 2018; Wang et al., 2020b). Therefore, the cinnamaldehyde-induced activation of Nrf2 may also be responsible for the reduced ROS production and subsequent NLRP3 inflammasome inhibition.

Both cinnamaldehyde and astaxanthin (**27**) are effective at alleviating organ fibrosis by repressing oxidative stress. In rats fed with fructose, cinnamaldehyde represses cardiac fibrosis by supressing oxidative stress and ROS production, thereby inhibiting NLRP3 inflammasome activation *via* the CD-36-mediated TLR4/6-IRAK4/1 signaling pathway (Kang et al., 2016). In a doxorubicin-induced mouse model of focal segmental glomerulosclerosis, astaxanthin elicits significant improvements in glomerular and interstitial fibrosis by promoting Nrf2 expression and inhibiting the NLRP3 inflammasome (Liu et al., 2015).

## Crude Extracts That Inhibit NLRP3 Inflammasome Activation and Improve Fibrosis

Relatively few crude extracts obtained from natural plants have been reported to exhibit antifibrotic activity *via* NLRP3 inflammasome inhibition and mainly include salvianolate (Sal), Danggui Buxue Tang (DBT), *Quamoclit angulata* (QA), and Huangkui capsule (HKC) (Table 2).

**TABLE 2:** Crude extracts from Traditional Chinese Herbs showed to modulate NLRP3 inflammasome activation in various fibrotic diseases.

Crude extracts	Model System	Potential mechanism
Salvianolates (Qiu et al., 2018)	Sprague-Dawley	Reducing the levels of IL-1β and IL-18 and escalating the expression of SIRT1 through TXNIP/NLRP3 pathway
Danggui Buxue Tang (Wang et al., 2016)	Sprague-Dawley	Suppressing NLRP3 inflammasome activation
Quamoclit angulata (Park et al., 2020)	C57BL/6	Inhibiting NLRP3 inflammasome-dependent pathway
Huangkui capsule (Han et al., 2019)	Sprague-Dawley	Depressing NLRP3 inflammasome and TLR4/NF-κB signaling activation

Sal, the major water-soluble bioactive fraction of the *Salvia miltiorrhiza*, is composed of lithospermate B, rosmarinic acid, and lithospermic acid. In postmyocardial infarction (MI) model rats, Sal effectively reduces the left atrial levels of IL-1β and IL-18 and upregulates the expression of SIRT1, an enzyme that suppresses the TXNIP/NLRP3 inflammasome signaling pathway, thereby alleviating atrial interstitial fibrosis (Qiu et al., 2018).

DBT is a traditional Chinese medicine composed of two herbs, namely, Radix Astragali and Radix Angelicae Sinensis at a ratio of 5:1 (Gao et al., 2011). Studies have shown that total glucosides of DBT repress bleomycin-induced pulmonary fibrosis (Gao et al., 2011). In rats with unilateral ureteral obstruction (UUO), DBT treatment significantly reduces tubulointerstitial collagen deposition and tubulonterstitial fibrosis *via* suppressing NLRP3 inflammasome activation, thus protecting the rats with UUO (Wang et al., 2016).

QA is used in the treatment of diabetes and associated complications. In a mouse model of type 2 diabetes mellitus (T2DM), QA extract effectively attenuates renal fibrosis in a NLRP3 inflammasome-dependent manner (Park et al., 2020).

HKC, extracted from the *Abelmoschus manihot* flower, is a modern Chinese patent medicine composed mainly of flavonoids that is used for the treatment of chronic kidney diseases (Cai et al., 2017). In rat models of early DN, HKC significantly alleviates EMT effects by suppressing NLRP3 inflammasome and TLR4/NF-κB signaling activation in the kidneys (Han et al., 2019).

## Conclusion and Future Perspectives

NLRP3 is a major sensor of the innate immune system, the aberrant activation of which contributes to various fibrotic diseases, including NASH, CKD, DN, and idiopathic pulmonary fibrosis (IPF). Identifying NLRP3 inhibitors in plant-derived natural products is of great importance for drug discovery as they are mostly safe, extensively distributed, and widely available. Among all the compounds, the diterpenoids may be the most promising candidates, for they possess combined inhibitory effect on NF-κB (the first signal of NLRP3 activation) pathway and NLRP3 inflammasome assembly (Hortelano et al., 2020). In this review, we have only summarized the natural products for which there is direct evidence that their antifibrotic effects are exerted *via* suppression of the NLRP3 inflammasome. Although other natural products, such as salidroside, berberine, and astragaloside IV, exhibit both NLRP3 inhibitory activity and antifibrotic activity (Dai et al., 2017; Dinesh and Rasool 2017; Guan et al., 2018; Qian et al., 2018; Li et al., 2019; Zhang et al., 2020), further investigation is needed to reveal whether the relationship is direct. In addition, the natural products currently reported to improve fibrogenesis through inhibiting NLRP3 inflammasome activation can only ameliorate fibrosis from early stages and cannot reverse existing fibrosis. Finally, in view of the NLRP3 inhibitors described above, further exploration is required for the following aspects. Firstly, although the binding sites for several natural products in NLRP3 inflammasome-related proteins have been identified, further studies are needed to reveal the mechanisms of action of potential NLRP3 inflammasome inhibitors and identify molecules that can selectively antagonize NLRP3 for the treatment of fibrotic diseases. Secondly, as a wide variation in the dose of compound used in different research may result in off-target effects and false-positive results, standardization of the dose for specific inhibition of NLRP3 will be of great significance for future studies. Thirdly, more efforts should be paid to the toxicity of the compounds *in vivo*, as unclear causes of side effects markedly restrict their application in clinic.

## Author Contributions

ND, BW, and XF prepared the draft. All the authors modified the draft.

## Funding

This work was supported by the National Natural Science Foundation of China under Grant Nos. 81671986 and 31872643, Hunan Provincial Key Laboratory for Special Pathogens Prevention and Control Foundation under Grant No. 2014-5, and the Hunan Province Cooperative Innovation Center for Molecular Target New Drug Study (2015-351).

## Conflict of Interest

The authors declare that the research was conducted in the absence of any commercial or financial relationships that could be construed as a potential conflict of interest.
